# Mapping the emerging burden of dengue

**DOI:** 10.7554/eLife.47458

**Published:** 2019-05-13

**Authors:** Oliver Brady

**Affiliations:** 1 Department of Infectious Disease EpidemiologyLondon School of Hygiene & Tropical MedicineLondonUnited Kingdom; 2 Centre for the Mathematical Modelling of Infectious DiseasesLondon School of Hygiene & Tropical MedicineLondonUnited Kingdom

**Keywords:** seroprevalence, Bangladesh, dengue, nationally-representative, Virus

## Abstract

The first nationally-representative survey of dengue has revealed the growing burden of the disease in Bangladesh.

**Related research article** Salje H, Paul KK, Paul R, Rodriguez-Barraquer I, Rahman Z, Alam MS, Rahman M, Al-Amin HM, Heffelfinger J, Gurley E. 2019. Nationally-representative serostudy of dengue in Bangladesh allows generalizable disease burden estimates. *eLife* **8**:e42869. doi: 10.7554/eLife.42869

Dengue is a viral disease spread by mosquitoes and found in more than 120 countries around the world (Bhatt et al., 2013). Because the species that transmits the disease, *Aedes aegypti*, lives in dense man-made environments, recent urbanization throughout the tropical world has accelerated the spread of dengue (Bhatt et al., 2013). *Aedes aegypti* is also the primary vector for a number of other diseases, including Zika, chikungunya and Yellow Fever, so understanding how to control the global emergence of dengue could help to prevent the spread of these diseases.

However, getting accurate data on who is being infected is surprisingly difficult. Only a small proportion of people (11–32%) are likely to have symptoms after contracting dengue, with few being sick enough to require formal medical care (Bhatt et al., 2013; Undurraga et al., 2013). And even if they receive medical care, misdiagnosis and under-reporting are common. This means that maps based on clinical case counts are not always useful, and may just reflect differences in access to healthcare, diagnostics and ability to report cases.

The gold standard for measuring who has been infected with dengue is a seroprevalence survey, where serum (blood) samples are taken from a representative group of people and tested for antibodies against the dengue virus. Now, in eLife, Henrik Salje and colleagues (who are based at institutes in France, the United States and Bangladesh) report how they have used this approach, which is usually reserved for local studies, on a scale never seen before to provide new insights into the transmission of dengue at a national level in Bangladesh (Salje et al., 2019).

Salje et al. visited 70 randomly selected communities across Bangladesh, between 2014 and 2016. During these visits, randomly selected residents were asked to contribute a blood sample and to answer a range of questions about behaviours thought to increase or decrease the risk of dengue. Overall, they found that 24% of participants showed evidence of previous dengue infection: this was much lower than the figures in other affected regions, such as Indonesia (~70%), where dengue has been around much longer (Tam et al., 2018). They also found that males were more likely to be infected than females, and that regular travellers were almost twice as likely to be infected as people who did not travel regularly.

However, the biggest factor in determining the risk of infection was location: over 80% of individuals in big cities, such as Dhaka, had been infected with the virus at some stage in their life, whereas fewer than 5% of people in rural areas showed signs of any previous infection. Using the results of their survey, Salje et al. were able to construct models that could predict seroprevalence in areas where data had not been collected, and then use this information to produce an updated risk map for dengue across Bangladesh (Figure 1).

**Figure 1.**
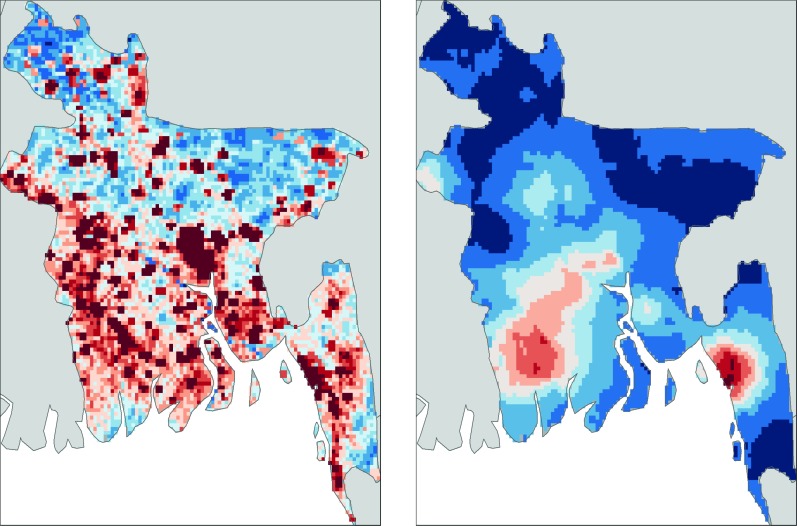
Mapping dengue risk in Bangladesh. A cross-sectional seroprevalence map (right) of dengue risk in Bangladesh, based on data collected from 70 communities throughout the country (Salje et al., 2019), shows that the risk is highest in the cities of Chittagong (in the south east), Khulna (south west) and Dhaka (in the middle of the country). Previous maps, such as this map based on data from Bhatt et al. (2013) (left), have predicted more widespread risk throughout the country, but these maps were largely based on less detailed data and often relied on extrapolation from nearby countries. This new study by Sajle et al. may suggest that there are unique, as yet unknown, factors that constrain the current distribution of dengue in Bangladesh.

By comparing seroprevalence between different age groups, Salje et al. were able to estimate the rate at which infections accumulate. Using standard 'catalytic models' (and making some assumptions about how long dengue has been in Bangladesh) they estimated that about 2.4 million people, out of a population of ~160 million, are infected with dengue virus in Bangladesh each year. In contrast fewer than 6,000 dengue cases per year were reported over the same time period, mostly in Dhaka (Mutsuddy et al., 2019). The results from this survey have the potential to play an important role in expanding and adapting dengue surveillance practices in Bangladesh.

Salje et al. also suggest that the current distribution of mosquito species in Bangladesh may be the cause of the limited spread of dengue. The mosquito that carries dengue, *Aedes aegypti*, was found in higher abundance in urban areas, whilst *Aedes albopictus*, a mosquito species that carries and transmits the virus less effectively, was more common in rural areas.

Together, these results paint a picture of a country part way through the emergence of dengue. Transmission is already high in urban centres but – unlike many countries in South East Asia and Latin America – regular, continuous transmission has yet to spread to more rural areas. However, more work is needed to understand how human movement and lower transmission potential in rural areas interact in shaping the current distribution of dengue in Bangladesh (Wesolowski et al., 2015). Genetic analyses of circulating dengue viruses can be used to identify the origins of outbreaks (Salje et al., 2017), which could help with efforts to contain the spread of the virus.

By proving the feasibility and value of nationally-representative seroprevalence surveys for dengue, it is hoped that the results of Salje et al. will renew interest in mass disease surveillance for dengue. Such community-representative surveys have proven instrumental for targeting and evaluating interventions for successful malaria control (Bhatt et al., 2015). A range of new dengue vaccines are currently becoming available, but some of these will only be beneficial if targeted to the areas of highest transmission (Flasche et al., 2016). Therefore, having regularly updated maps that accurately show the national spread of dengue will be critical in the ongoing fight against further expansion of the disease.
